# Psychological Vulnerability and Early Experiences as Predictors of Low Self-Perception in Patients with Eating Disorders

**DOI:** 10.1192/j.eurpsy.2026.11379

**Published:** 2026-07-31

**Authors:** G. Cano-Escalera, A. Flores, M. Ramirez, I. Gómez Grávalos, M. Melero González, I. Zorrilla, A. Gonzalez-Pinto

**Affiliations:** BIORABA, Department of Psychiatry, Hospital Universitario de Alava, CIBERSAM, UPV/EHU, Vitoria, Spain

## Abstract

**Introduction:**

Low self-esteem is a central feature of Eating Disorders (EDs), influencing both the onset and persistence of symptoms. Several studies point to the importance of individual factors (anxiety, depression), clinical factors (eating symptoms), and contextual factors (early adverse experiences) in self-perception. However, the evidence is heterogeneous, and the specific weight of these variables has not always been analyzed jointly. Understanding which factors predict low self-perception in patients with eating disorders is essential for designing more effective and personalized interventions.

**Objectives:**

To identify, using logistic regression, the most robust predictors of low self-perception in patients with eating disorders.

**Methods:**

A sample of patients diagnosed with eating disorders was evaluated. Self-perception was measured using the Rosenberg Scale, classifying participants as having low or normal self-esteem. Questionnaires on eating symptoms (EDI, EAT-40), anxiety (STAI), depression (Hamilton), adverse childhood experiences (Childhood Trauma Questionnaire), and additional clinical variables (BMI, cognitive tests) were administered. Binary logistic regression was applied to determine predictors of low self-esteem.

**Results:**

The binary logistic regression model showed a significant fit (Δχ^2^ = 28.213, p < .001), explaining between 56% and 72% of the variance in low self-esteem (Nagelkerke R^2^ = 0.724).

The significant predictors were:

- Eating symptoms (EDI_TOTAL): β = -0.004, p = .003; OR ≈ 0.996 indicating a robust association
- Trait anxiety (STAI-R): β = 0.331, p = .050; OR ≈ 1.39.
- Adverse childhood experiences (Childhood_TOT): β = 0.129, p = .037; OR ≈ 1.14.

Eating disorder symptoms, state anxiety, depression (Hamilton), and CTQ did not reach significance in the model.

**Conclusions:**

Logistic regression identified three key factors predicting low self-perception in eating disorders: eating symptoms, trait anxiety, and adverse childhood experiences. These results suggest that self-esteem in eating disorders is explained by the interaction of current clinical factors (eating symptoms), stable emotional vulnerabilities (trait anxiety), and early life conditions (childhood trauma). Addressing these three levels simultaneously can improve therapeutic efficacy and promote comprehensive recovery.

**Disclosure of Interest:**

None Declared

